# Climate Change Simulations Predict Altered Biotic Response in a Thermally Heterogeneous Stream System

**DOI:** 10.1371/journal.pone.0111438

**Published:** 2014-10-30

**Authors:** Jacob T. Westhoff, Craig P. Paukert

**Affiliations:** 1 Missouri Cooperative Fish and Wildlife Research Unit, Department of Fisheries and Wildlife Sciences, University of Missouri, Columbia, Missouri, United States of America; 2 U.S. Geological Survey, Missouri Cooperative Fish and Wildlife Research Unit, University of Missouri, Columbia, Missouri, United States of America; Clemson University, United States of America

## Abstract

Climate change is predicted to increase water temperatures in many lotic systems, but little is known about how changes in air temperature affect lotic systems heavily influenced by groundwater. Our objectives were to document spatial variation in temperature for spring-fed Ozark streams in Southern Missouri USA, create a spatially explicit model of mean daily water temperature, and use downscaled climate models to predict the number of days meeting suitable stream temperature for three aquatic species of concern to conservation and management. Longitudinal temperature transects and stationary temperature loggers were used in the Current and Jacks Fork Rivers during 2012 to determine spatial and temporal variability of water temperature. Groundwater spring influence affected river water temperatures in both winter and summer, but springs that contributed less than 5% of the main stem discharge did not affect river temperatures beyond a few hundred meters downstream. A multiple regression model using variables related to season, mean daily air temperature, and a spatial influence factor (metric to account for groundwater influence) was a strong predictor of mean daily water temperature (r^2^ = 0.98; RMSE = 0.82). Data from two downscaled climate simulations under the A2 emissions scenario were used to predict daily water temperatures for time steps of 1995, 2040, 2060, and 2080. By 2080, peak numbers of optimal growth temperature days for smallmouth bass are expected to shift to areas with more spring influence, largemouth bass are expected to experience more optimal growth days (21 – 317% increase) regardless of spring influence, and Ozark hellbenders may experience a reduction in the number of optimal growth days in areas with the highest spring influence. Our results provide a framework for assessing fine-scale (10 s m) thermal heterogeneity and predict shifts in thermal conditions at the watershed and reach scale.

## Introduction

The ecological importance of water temperature to aquatic organisms has been the impetus for numerous studies that sought to develop predictive temperature models for various systems [1]–[3]. Many external drivers interact with the physical properties of rivers to determine water temperature and include air temperature, solar radiation, relative humidity, wind speed, riparian shade, cloud cover, solar angle, discharge, tributary contributions, and groundwater contributions [4]–[6]. However, it is often not feasible to obtain information on all of these factors for an aquatic system of interest, especially at the fine spatial scales required to document thermal heterogeneity. Lotic systems heavily influenced by groundwater inputs create spatially heterogeneous thermal environments and are difficult to explain with coarse-scale temperature models [7], [8]. Progress has been made to address the heterogeneity of stream water temperatures at finer spatial scales [9]–[12], but collecting appropriate data to parameterize models that can be applied over long distances (100 s km) at a fine-scale spatial resolution (10 s m), while accounting for seasonal variation, is difficult.

Groundwater springs occur in patchy distributions around the globe and provide unique physical and chemical environments that support many biological assemblages [13], [14]. Systems with significant groundwater input or cold-water tributaries serve as thermal refuges for aquatic species [15]–[20]. Further, certain species can exist in groundwater fed systems that may not be able to survive in geographically proximate systems lacking groundwater influence [21], [22]. Species composition within and outside of springs is also known to differ, enhancing beta diversity in the system [14]. At a coarse spatial scale, groundwater can influence the distribution and abundance of aquatic organisms [23], [24] and reduce the occurrence of temperature fluctuations that may result in reproductive failure of certain species [25]. At fine spatial scales, some fishes show behavioral responses to thermal refuges by selecting spawning locations [26], [27], avoiding ice break up and frazil ice [28], or thermoregulating by occupying groundwater influenced areas during warm or cool water periods [16], [19], [20], [29].

Climate change has the potential to alter environmental conditions in streams in many ways, but especially the physical properties associated with discharge and water temperature [30], [31]. Altered environmental conditions in aquatic systems may result in physiological effects [32], [33], behavioral or competitive effects [34], [35], or shifts in the distribution and abundance of aquatic organisms [36]–[38]. Some of the effects of climate change may be buffered in thermally heterogeneous stream systems with high levels of groundwater influence [28], [39]; however, little information exists linking predicted water temperatures to thermal requirements of aquatic organisms in these systems [40], [41]. Climate change is frequently listed as a threat to groundwater-dependent biota, but direct quantification of potential effects is less common [42], [43]. Efforts to predict climate change effects on thermal conditions in streams at a regional scale often do not include predictive variables that specifically account for groundwater influence, especially at fine-spatial scales, which can result in models with limited inference for heavily groundwater influenced systems [7], [9], [44]. Because of the importance of groundwater influenced systems, it is important to quantify their dynamics and how they might respond to a changing climate so that managers tasked with protecting biodiversity in the face of climate change proceed effectively [45], [46].

Our goal was to develop an approach that could be used to model water temperature in groundwater dominated lotic systems that are of high conservation importance and do not conform with coarse-scale temperature modeling approaches. Further, we wished to explain thermal heterogeneity within a mainstem river system heavily influenced by groundwater inputs and predict how biota of high conservation concern may respond. To achieve these goals, we addressed four main objectives: 1) document longitudinal variation in stream temperature at the warmest and coldest times of the year, 2) create a spatially-explicit temperature model based on empirical data to predict daily average temperature, 3) apply the predictive model to forecast the effects of two climate change scenarios on water temperature, and 4) link predicted water temperatures based on climate change simulations to three aquatic organisms of concern to conservation and management but that have different temperature requirements.

## Methods

### Study Location

**Figure 1:**
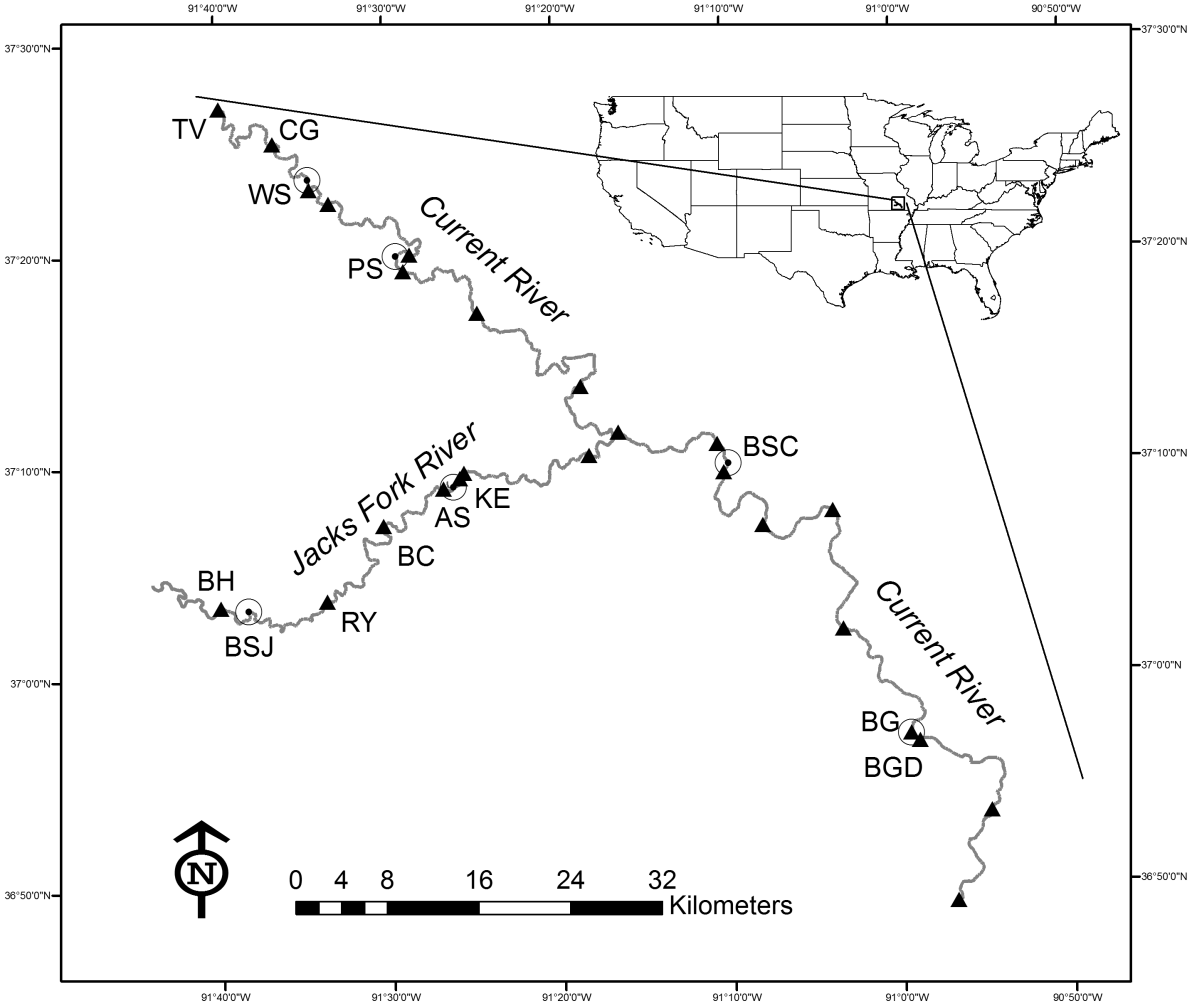
Location of the Ozark National Scenic Riverways (Jacks Fork and Current Rivers). Triangles indicate locations of stationary temperature loggers deployed for the entirety of 2012, with locations mentioned in the text noted by abbreviations (Tan Vat, TV; Cedar Grove, CG; Big Spring Downstream, BGD; Buck Hollow, BH; Rymers, RY; Bay Creek, BC, and Keatons, KE). Hollow circles surrounding a point indicate the locations of major springs referenced in the text (Welch Spring, WS; Pulltite Spring, PS; Blue Spring Current, BSC; Big Spring, BG; Blue Spring Jacks Fork, BSJ; and Alley Spring, AS).

Our study occurred within the Ozark National Scenic Riverways (ONSR), which is a National Park Service Unit located in south-central Missouri, USA (37° N, 91° W) on the Ozark Plateau [47]. The park encompasses approximately 32,700 hectares, creating a narrow corridor along 215 km of the Current River and its largest tributary, the Jacks Fork River (Figure 1). The Current River is a southerly flowing stream which enters the ONSR as a 4^th^ order [48] stream and reaches 6^th^ order upon its exit, whereas the Jacks Fork River is an eastern flowing 5^th^ order stream within the ONSR. Average (range) wetted channel width in the Current River was 47.6 m (17.5 – 127.3 m) and 26.3 m (12−49 m) in the Jacks Fork (J. Westhoff, unpublished data). The deepest pools in the Current and Jacks Fork Rivers rarely exceed 5 and 3 m, respectively (J. Westhoff, unpublished data). Substrate composition in the river channel was generally dominated by coarse chert gravel or large boulders associated with bluff pools or high gradient reaches [49]. The riparian zone was dominated by deciduous forest and was mostly intact along the entirety of the river contained with the ONSR. The overall catchment was primarily forested with 14% of the catchment in cleared land, only 2% of which is on areas with > 7° slopes [50].

The ONSR is characterized by deep valleys overlaying karst topography, which creates many caves and springs. Big Spring, one of the largest springs in the world, is located on the Current River and has an estimated annual mean discharge of 12.6 m^3^/sec [47]. Many other large springs exist along the Current and Jacks Fork Rivers (Appendix S1) and groundwater sources account for over 90% of the total discharge within the ONSR [47]. Throughout the remainder of the manuscript, the acronym “ONSR” will refer only to the mainstem Current and Jacks Fork Rivers within the park. Field research for this study was completed under permit OZAR-2011-SCI-0007 from the United States National Park Service.

### Data Collection

Longitudinal temperature surveys were conducted by boat during winter (Jan 18 – Feb 23, 2012) and summer (July 30 – Aug 15, 2012) over the entire ONSR. Temperatures were obtained over multiple days during daylight hours and in different sections of the ONSR from 10 – 25 km long each day, depending on river access locations. For the winter survey, temperature was recorded using an Aqua Troll 100 (In-Situ Inc., Fort Collins, CO; accuracy ±0.1°C) by recording temperature approximately 10 cm below the water surface every 30 seconds while moving in a downstream direction. Temperature values were spatially linked using a GPS (Archer Field PC with Hemisphere GPSXF101, Juniper Systems Inc., Logan UT) with time settings synced to the Aqua Troll 100 device to record UTM coordinates every 30 seconds. During summer, water temperatures were taken 10 cm below the water surface every 250 m along the ONSR using an YSI 550A (YSI Inc., Yellow Springs, OH; accuracy ±0.3°C) and linked with UTM coordinates.

Longitudinal surveys conducted in the winter and summer captured spatial variation in temperature over the entire ONSR, whereas temporal variation in temperature was captured using stationary temperature loggers (HOBO Pendant, Onset Computer Corp., Cape Cod, MA; accuracy ±0.53°C). Loggers were installed at 26 locations throughout the ONSR and were generally located within a few hundred meters of an established river access location or just above and below major groundwater inputs (Figure 1 and Appendix S2). The average (± Std. Dev) distance between loggers was 9.3 ± 5.6 km. Temperature logger installation and removal dates varied, but 25 loggers recorded data every 30 minutes throughout calendar year 2012 (Appendix S2).

### Longitudinal Water Temperature Variation Analysis

The use of three different temperature recording devices necessitated correction of temperature readings. Laboratory derived correction factors resulted in the addition of 0.1°C and 1.0°C to raw temperature values for the Aqua Troll 100 and YSI 550A, respectively, to achieve standardization with the HOBO Pendant loggers. Longitudinal temperature survey data were collected at various times of day and on different days, which required correction of values and standardization to the same moment in time for valid comparisons. Therefore, we used data collected from stationary temperature loggers to address issues of both temporal and spatial standardization. We collected information on the closest upstream and downstream temperature loggers for each longitudinal survey point, the distance to those loggers, and the temperature of those loggers at the time the survey point occurred. The distances from the survey point to each logger were used to create a weight for the logger specific to each survey point based on inverse distance weighting (IDW), where a factor of −2 was used for the exponent [51]. The difference in temperature between expected and observed values based on interpolation of temperature logger values was termed the spatial correction factor (SCF) and was calculated with the following equation,
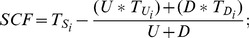
Where *Ts_i_* was the temperature recorded at survey point *i*, *U* was the weight of the closest upstream logger to point *i*, *D* was the weight of the closest downstream logger to point *i*, *Tu_i_* was the temperature recorded at the closest upstream logger at the time when temperature was recorded at survey point *i*, and *Td_i_* was the temperature recorded at the closest downstream logger at the time when temperature was recorded at survey point *i.*

Next, to standardize all the measured temperature readings to a single point in time, the weighting process was repeated using the extreme (either minimum or maximum, depending on season of survey) temperature values for the two loggers closest to the survey point. The mode of the coldest day and time observed (January 14, 2012 at 0830 hours) and the mode of the warmest day and time observed (July 25, 2012 at 1700 hours) were selected from the 25 stationary logger datasets and the values for those days served as the values to which all others would be standardized. This weighting provided a predicted high or low temperature value for that survey point location which was then added to the spatial correction factor. These final values represented temporally and spatially corrected temperature values for every survey point for the coldest and warmest time of the year. If a major groundwater feature entered the system, the IDW methodology varied in that the nearest logger may not have been used (e.g., a point just downstream of a major spring would have none of its weight based on a logger above the spring).

### Relation of Spring Magnitude to Spring Influence

Each spring in a system contributes a different percentage of discharge to the total river discharge [47] and affects the water temperature for various distances downstream [47], [52]. This relationship between spring discharge and distance from spring is complex, especially when considered in conjunction with other spatially varying factors that affect water temperature [5]. Knowledge of individual thermal contribution from a given spring to the river was investigated based on observed longitudinal temperature variation. We selected nine springs that contributed at least 5% of the river discharge and used information obtained from our summer maximum and winter minimum estimates to determine the relationship between spring magnitude (M) and spring influence (I). Spring magnitude was defined as the discharge of the spring divided by the discharge of the river at its confluence with the spring and calculated with data from Mugel et al. [47]. Spring influence was calculated for summer (*SI_Max_*) and winter calculated winter (*SI_Min_*) conditions using the corrected temperature values described in the previous section (maximum or minimum corrected temperatures) following the equations;
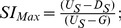
and



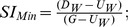
where U_S_ is the temperature upstream of a given spring in the summer, U_W_ is the temperature upstream of a given spring in the winter, D_S_ is the temperature downstream of a given spring in the summer, D_W_ is the temperature downstream of a given spring in the winter, and G is the temperature of the spring water (14°C was used in our analysis). This allowed us to display the spring influence relative to how much temperature change was possible given the difference between river temperature and groundwater temperature. Spring influence values of 0 indicated no influence and a value of 1 indicated complete spring influence. Linear regression was used to determine the relationship between spring magnitude and spring influence for both summer and winter conditions.

### Predictive Water Temperature Modeling

We used multiple regression to create a predictive water temperature model for the main stem Jacks Fork and Current Rivers within the ONSR. We chose a statistical approach because of data availability and concerns about addressing the spatial variation caused by groundwater influence. Statistical models are based on correlations among water temperature, air temperature, and other factors and often have less comprehensive data requirements than deterministic models [6], [53]–[55]. Deterministic models rely on the physical properties of water and heat exchange and require large amounts of meteorological and hydrological data [56], [57].

Because the system was greatly influenced by groundwater, we standardized all air and water temperatures based on the average temperature of groundwater entering the system. Our approach follows the equilibrium temperature concept described by Mohseni & Stefan [37] which identified the point in time when no heat is transferred between air and water; however, we instead focused on heat transfer between groundwater and river water. Instead of estimating heat flux using data on radiation, evaporation, and other physical properties of heat exchange in water, we assumed a constant groundwater temperature of 14°C and subtracted that value from observed air and water temperature values to create a linear transformation of the response of water temperature to any groundwater input. Discharge was not included as a predictor in the model, but was controlled for by removing any observations from days when discharge exceeded the 75% percentile of records for the closest USGS gage station.

We accounted for spatial influences on water temperature by creating a spatial influence factor (SIF). Numerous spatial drivers of water temperature (e.g., stream size, land use, riparian coverage) were accounted for using the SIF, but the relative effects of any one driver were unknown. However, the primary spatial driver in this system captured by the SIF was groundwater influence. The SIF was calculated based on results from the longitudinal temperature survey conducted in the summer of 2012 and the resulting spatial correction values. The corrected temperature at each survey point along the longitudinal transect was subtracted from the warmest temperature that occurred in the ONSR (31.9°C). Resulting values were then divided by the greatest observed difference in temperature between any two points in the system (13.7°C) to standardize them for the ONSR. Resulting values ranged from 0 (no spring influence) to 1 (greatest spring influence). We determined the SIF values for every river reach in the Jacks Fork and Current Rivers to determine the composition of SIF values within the ONSR. The SIF value differs from the spring influence value because it incorporates all spatial drivers of water temperature and can be determined for any location on the river, not just below a spring.

Air temperature is a strong predictor of water temperature in lotic systems, but generally performs best when considered as a moving average as opposed to an instantaneous value congruent with the water temperature reading [58]. Thus, we used five day average daily air temperature (AirTemp) where the average daily air temperatures on the day of the reading and the four previous days were averaged using weighting factors. Daily air temperatures were averaged by multiplying the average temperature on the day of the observation by 0.3, the prior day by 0.3, the next previous day by 0.2, and the other two previous days by 0.1. All air temperature data were from the Round Spring weather station which was centrally located in the ONSR and within 70 km of the furthest location on the river to which the model applies.

**Figure 2:**
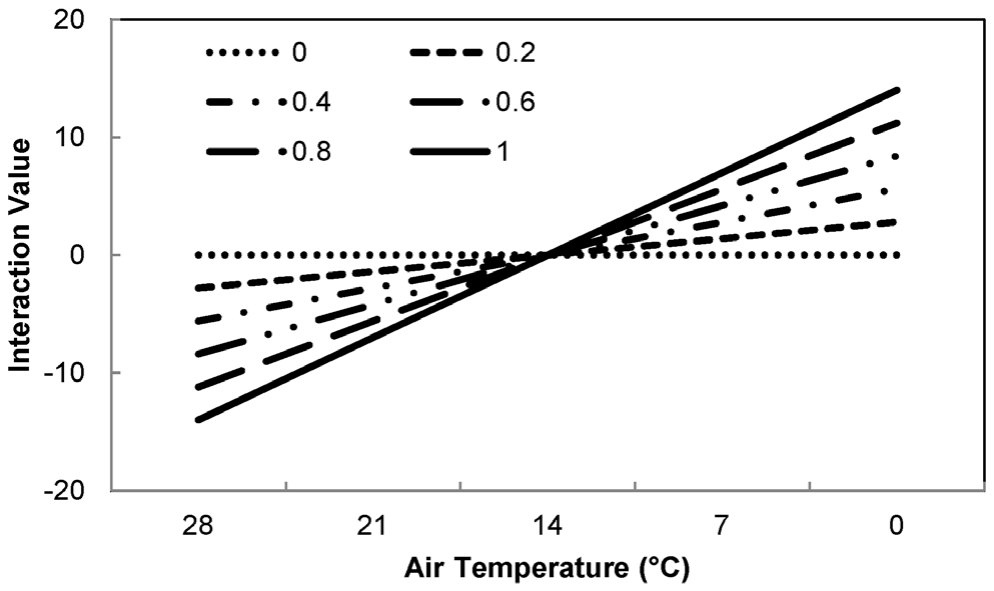
Simulated values of the interaction term when spring influence is multiplied by season influence under six levels of spring influence (0, 0.2. 0.4. 0.6, 0.8, and 1; 0 = no spring influence, 1 = heavy spring influence). Results are displayed by unadjusted air temperature for clarity, but air temperature values used in calculations were subtracted from 14°C.

Finally, we accounted for seasonal effects on water temperature with a metric (Season) based on climate normals and how they related to groundwater temperature. Climate normals [59] were obtained from the West Plains, MO weather station (40 – 80 km southwest of ONSR) as opposed to the Round Springs weather station because the data record had fewer missing values and a longer record. The average daily air temperature value for each calendar day was subtracted from 14°C; thus, the metric had a value of zero when air temperatures were equal to groundwater temperatures (Mid April and Mid October). Days with average temperatures above 14°C had negative values and days below 14°C had positive values, which mimicked the effect of groundwater input on the ambient water temperatures. The interaction between season and the SIF was of interest because multiplying the SIF by the groundwater influence resulted in season values that were essentially weighted by the amount of spring influence (Figure 2). This interaction accounted for both the direction and magnitude of the effect of groundwater influence at a given air temperature.

Temperature data from each of 25 temperature loggers were summarized by determining the average daily water temperature for each calendar day of 2012. Those values were standardized to groundwater temperature (as described above) and served as the response variable in our models. Data from all logger locations over all days in 2012 were compiled into one dataset and records were removed if discharge exceeded the 75% quartile during that day at that location, or if data were corrupt or missing. From the reduced dataset, 10% of the records were randomly selected to serve as training data for the models.

We used multiple regression techniques to develop a temperature model that predicted daily average water temperatures throughout the ONSR. Combinations of predictor variables were used to create six candidate models, which were compared based on their Akaike Information Criterion (AIC) values to select the best model (Table 1). These candidate models were chosen to examine the explanatory power of each of the variables by itself, and in combination. Model parameter estimates and intercepts were used along with the data withheld from the model creation set (90% of total observations) to validate the final model. All analyses were done in SAS 9.3 (Cary, NC).

**Table 1:** Candidate models used in multiple regression modeling to predict average daily water temperature in the Current and Jack’s Fork Rivers, Missouri.

Model Number	Model Structure	*AIC*	*w_i_*
1	β_0_ + β_1_(AirTemp)	1245.2	0
2	β_0_ + β_1_(Season)	1470.0	0
3	β_0_ + β_1_(SIF)	2823.6	0
4	β_0_ + β_1_(Season) + β_2_(SIF) + β_3_(Season*SIF)	807.7	0
5	β_0_ + β_1_(AirTemp) + β_2_(Season) + β_3_(SIF)	879.0	0
6	β_0_ + β_1_(AirTemp) + β_2_(Season) + β_3_(SIF) + β_4_(Season*SIF)	−329.2	1

The variable AirTemp represents a five-day weighted moving average air temperature. The variable Season represents a value based on climate normal air temperatures, and the variable SIF (spatial influence factor) represents a spatial variation in water temperature caused by groundwater and other factors. β_0_ represents intercept and β_1_ represents slope. Akaike Information Criterion (*AIC*) values and model weights (*w_i_*) are displayed for each candidate model.

### Climate Change Simulations and Predicted Biotic Implications

Simulated air temperature values for a central location within the ONSR were obtained from dynamically downscaled climate simulations [60] and incorporated into our predictive temperature model. Air temperature data (2-m above surface) were obtained from the downscaled versions of the MPI ECHAM5 (EH5) and GENMOM climate simulations [60]. These models were chosen because they provided data at a 15-km grid scale and to show a range of potential conditions within the A2 emissions scenario [60]. Air temperature estimates were averaged for each day of the year across five years (e.g., 2040 – 2044) for four time steps of 1995, 2040, 2060, and 2080. We calculated 5-day moving averages of the simulated air temperature values as outlined above, and substituted those values in our predictive model of average daily temperature in place of the AirTemp variable. We did not incorporate predicted changes in precipitation, discharge, or groundwater temperature based on climate simulations in our model. All other model inputs were identical to the original predictive temperature model.

We summarized forecasted results by applying ecologically important thermal criteria for three aquatic species that occur in the ONSR. Smallmouth bass *Micropterus dolomieu* and largemouth bass *Micropterus salmoides* are competitors [61], economically important sportfish [62], and possess different thermal tolerances and optimal growth temperatures (Table 2). The Ozark hellbender *Cryptobranchus alleganiensis bishopi* is a rare species of salamander listed by the United States Endangered Species Act. Temperatures at which these organisms no longer exhibit growth due either to cold or warm water temperatures, along with the optimal temperature range for growth, and the range for potential growth (Table 2) were used in our models. The number of days per year with average daily temperature within these ranges (final predicted water temperatures were rounded to the nearest whole digit) was summed for each simulated climate change scenario and across the range of spatial influence factor values. These biologically relevant temperature estimations allowed us to examine potential change in thermal suitability and bioenergetic response associated with climate change while accounting for spatial heterogeneity of stream temperatures.

**Table 2:** Ecologically important thermal criteria for smallmouth bass (*Micropterus dolomieu*), largemouth bass (*Micropterus salmoides*), and Ozark hellbender (*Cryptobranchus alleganiensis bishopi*) in the Ozark National Scenic Riverways.

Species	Positive growth (°C)	Optimal growth (°C)	Sources
Smallmouth bass	10−27	20−24	[52], [63]
Largemouth bass	15−36	24−30	[64]–[66]
Ozark hellbender	3−27	10−16	[67], consultation with experts

## Results

The greatest annual temperature variability (2.2 – 32.0°C) within the ONSR occurred at the upstream most location on the Jacks Fork River (Buck Hollow; Figure 1, Appendix S3), which is a location with very little groundwater influence. The lowest annual water temperature variation occurred at a site (Tan Vat) with a high degree of groundwater on the Current River, where water temperature ranged from 8.4 to 20.5°C. Alley Spring was the only location influenced entirely by groundwater with a temperature logger and ranged from 13.4 to 15.7°C with an average annual temperature (± standard deviation) of 14.2±0.4°C. Overall, average annual temperature ranged from 14.2°C (Tan Vat) to 17°C (Rymers and Bay Creek) within the ONSR (Figure 1, Appendix S3).

### Longitudinal Water Temperature Variation

**Figure 3:**
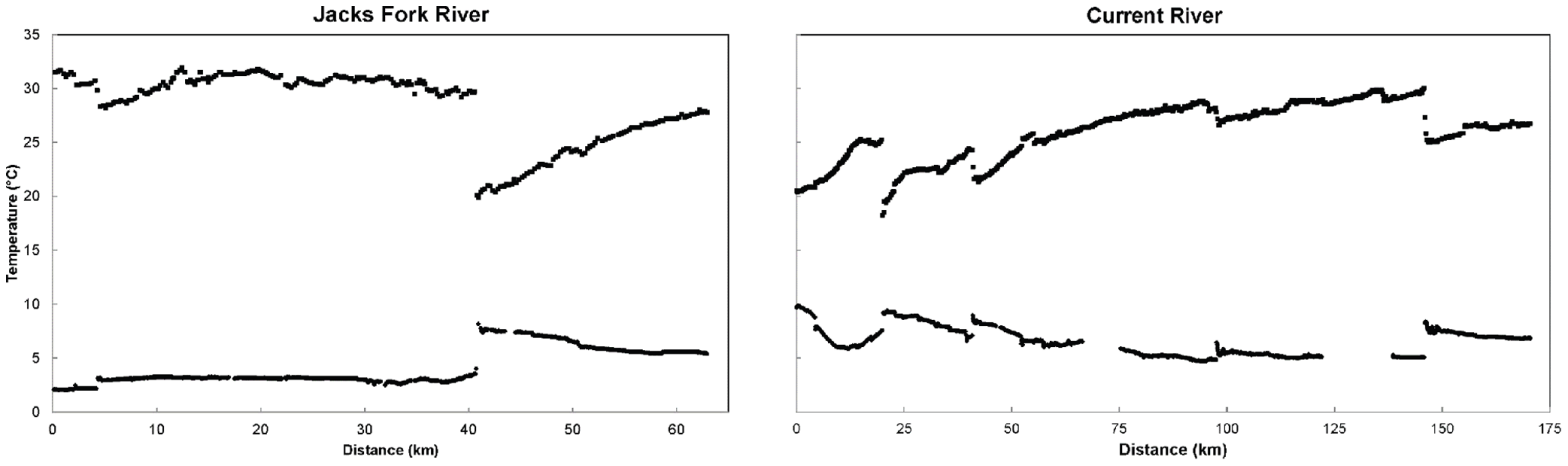
Maximum (upper dots) and minimum (lower dots) predicted temperatures along the Jacks Fork (left panel) and Current Rivers (right panel) during the warmest (July 25 at 5 pm) and coldest (January 14 at 8 am) periods of 2012. River distance for the Jacks Fork starts at the Buck Hollow access (0 km) and ends at the Current River confluence (63 km), with Blue Spring (4 km) and Alley Spring (41 km) accounting for major variation in temperature. River distance for the Current River starts at the Tan Vat access (0 km) and ends at the Gooseneck access (170 km), with Welch Spring (20 km), Pulltite Spring (40 km), Blue Spring (97 km), and Big Spring (146 km) accounting for major variation in temperature. Equipment malfunctions resulted in a loss of data during cold period sampling at two locations on the Current River (black boxes).

Spatial patterns in maximum and minimum temperatures observed from the longitudinal temperature surveys demonstrated the influence of groundwater input locations (Figure 3). The greatest effect of a single spring on the overall river temperature was observed downstream of Alley Spring on the Jacks Fork River, where summer maximum temperature decreased by approximately 10°C and winter minimum temperature increased by approximately 5°C (Figures 1 and 3). On the Current River, Welch Spring had the most influence on river temperature based on the 7°C decrease of maximum temperature and 3°C increase of minimum temperature (Figures 1 and 3). Other major springs that had greater than 1°C influence on stream temperature included Pulltite, Blue (Current), Big, and Blue Springs (Jacks Fork). Groundwater influence on river temperature directly downstream of springs was not as substantial in the winter as in the summer. Equipment malfunctions resulted in a loss of data for approximately 25 km of the Current River combined over two locations (River km 66 – 75 and 122 – 138) during the winter of 2012 (Figure 3).

### Relation of Spring Magnitude to Spring Influence

**Figure 4:**
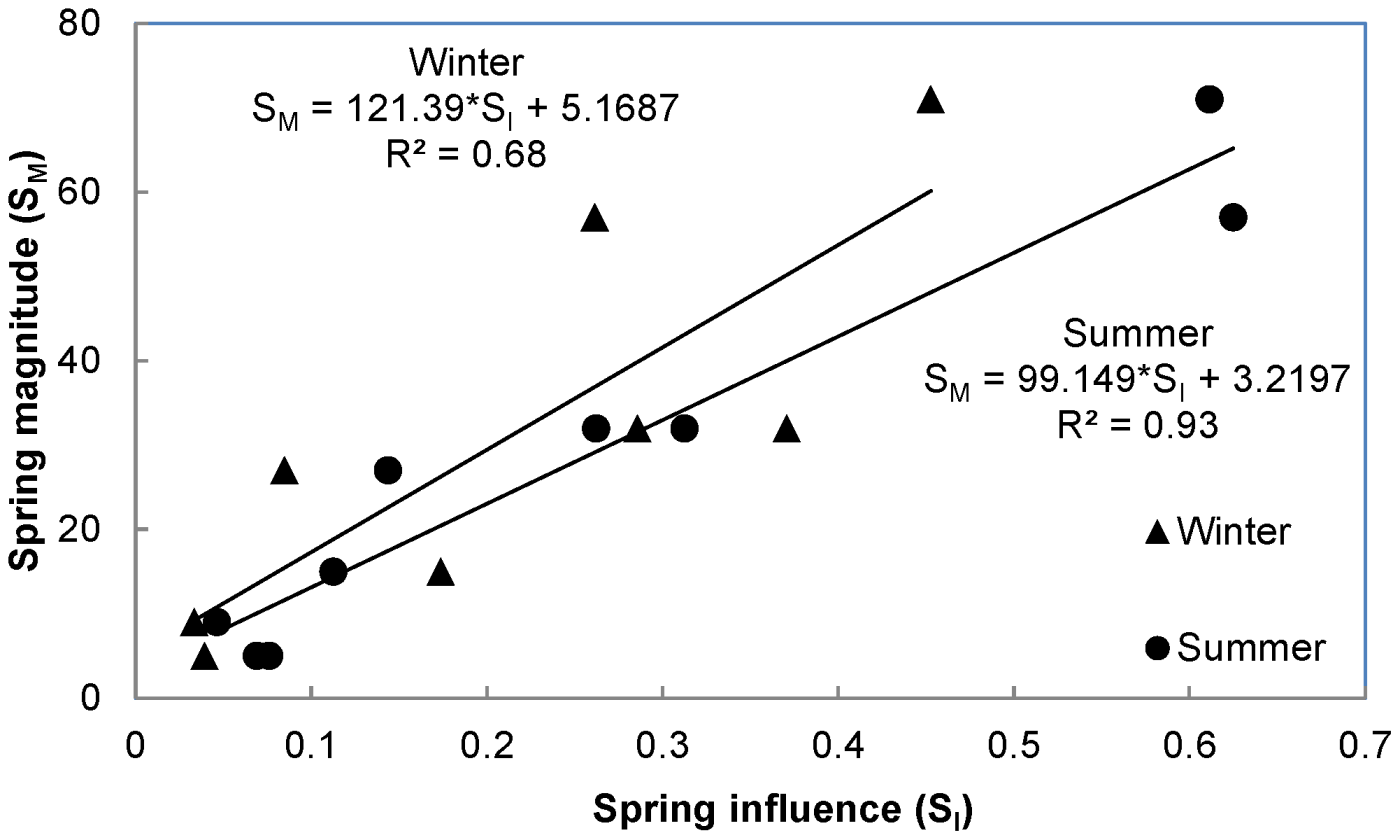
Relationship between spring magnitude (S_M_) and spring influence (S_I_) for both winter (triangles) and summer (circles) observations. Spring magnitude represents the percentage of discharge contributed to the river by the spring. Spring influence represents the percentage of change in water temperature from the upstream river temperature to groundwater temperature.

Spring influence on main stem river temperature was strongly related to spring magnitude during both summer (R^2^ = 0.93, intercept ± standard error = 3.2 ± 3.3, slope ± standard error = 99.1 ± 9.9) and winter (R^2^ = 0.68, intercept ± standard error = 5.2 ± 8.8, slope ± standard error = 121.4 ± 34.2); Figure 4). As spring magnitude increased, the influence of groundwater on river water temperature increased. Intercept values from the models indicated that a single spring contributing less than 3% of the total discharge in the summer or 5% in the winter would have no observable influence on river temperature at a location beyond 400 m downstream.

### Predictive Water Temperature Modeling

**Figure 5:**
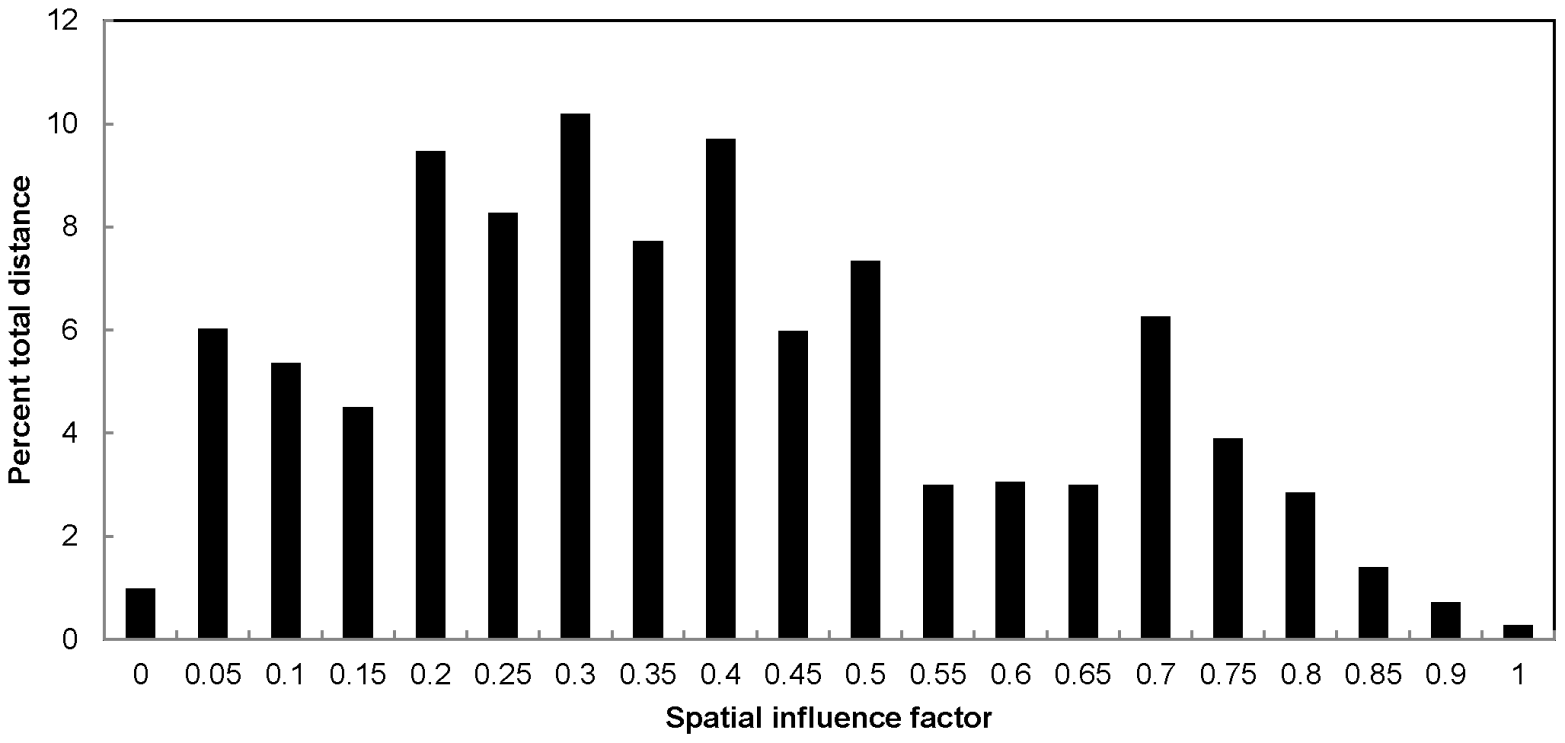
Percent of Jacks Fork and Current River distance within the Ozark National Scenic Riverways comprised by each of the spatial influence factor (SIF) values ranging from 0 to 1, by increments of 0.05.

We removed 547 temperature logger records (6%) because they exceeded the 75% quartile of discharge, with 95% of the removed records occurring between January 1 and March 26^th^. An additional 236 records (3%) were removed because they were corrupt or missing, resulting in 8367 valid records. The distribution of SIF values was uneven across the ONSR and had a distance weighted average SIF value of 0.40 (Figure 5). Approximately 75% of the ONSR had SIF values less than or equal to 0.50 (Figure 5). No model selection uncertainty existed as candidate model 6 (all variables and the Season*SIF interaction) had a model weight of one and indicated that the variables measured were more important than any single variable alone (Table 1). The average daily temperature model performed well based on R-squared (0.98) and RMSE (0.82) values, and compared favorably to other studies using RMSE as a validation metric [68], [69]. The final relationship to predict daily average water temperature (*WaterTemp*) was,
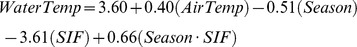



Associated standard error values for the parameter estimates were 0.05 (*Intercept*), 0.01 (*AirTemp*), 0.01 (*Season*), 0.11 (*SIF*), and 0.01 (Season*SIF). We observed high Pearson Correlation Coefficients between AirTemp and Season (−0.89 to −0.91) and between Season and SIF (0.85). All other Pearson Correlation Coefficients were below 0.80. None of the predictor variables had variance inflation factor (VIF) values >10, indicating that multicollinearity did not produce problems in our regression coefficients [70].

Model validation indicated the greatest underestimate of temperature observed was 3.3°C and the greatest overestimate was 3.4°C. The model predicted 98% of the observations within 2°C and 77% of the observations within 1°C. Estimated values 2°C warmer than observed came primarily (88%) from three sites (Buck Hollow, Cedar Grove, Tan Vat) and occurred in the summer (Figure 1). Estimates 2°C cooler than observed came primarily (82%) from two sites (Keatons and Big Spring Downstream) and occurred in the fall and winter (Figure 1).

### Climate Change Simulations and Predicted Biotic Implications

Average air temperature increased from the 1995 to 2080 time steps for all climate simulations (Table 3). The EH5 model predicted average air temperatures to increase by 2.8°C from 1995 to 2080, whereas the GENMOM model predicted a 2.1°C increase over the same time period. The average air temperatures at a given time for the EH5 model were commonly 2°C greater than those predicted by the GENMOM model. Mean yearly water temperatures were estimated to increase by 1.1°C from 1995 to 2080 based on the EH5 model, versus only 0.8°C for the GENMOM model (Table 3). Areas that were highly buffered by groundwater inputs were predicted to have 3.5°C cooler yearly average temperatures than areas with minor groundwater influence (Table 3). The 2040 and 2060 simulations were intermediate between the 1995 and 2080 simulations, so further discussion of the 2040 and 2060 results was omitted. However, 2040 simulations were similar to 1995 results and noticeable shifts in temperature became apparent by 2060.

**Table 3:** Summary temperature (°C) values for the Ozark National Scenic Riverways estimated using the MPI ECHAM5 (EH5) and GENMOM climate simulations at time steps of 1995, 2040, 2060, and 2080.

Simulation	Time-step	Average Air Temperature	Average Water Temperature(High SIF, Low SIF)
EH5	1995	12.2	13.3, 16.8
	2040	13.0	13.6, 17.1
	2060	14.2	14.1, 17.6
	2080	15.0	14.4, 17.9
GENMOM	1995	10.9	12.8, 16.3
	2040	11.2	12.9, 16.4
	2060	12.1	13.2, 16.8
	2080	13.0	13.6, 17.1

Low spatial influence factor (SIF) indicates areas with little groundwater influence (SIF = 0) and high SIF indicates areas with high groundwater influence (SIF = 1).

**Figure 6:**
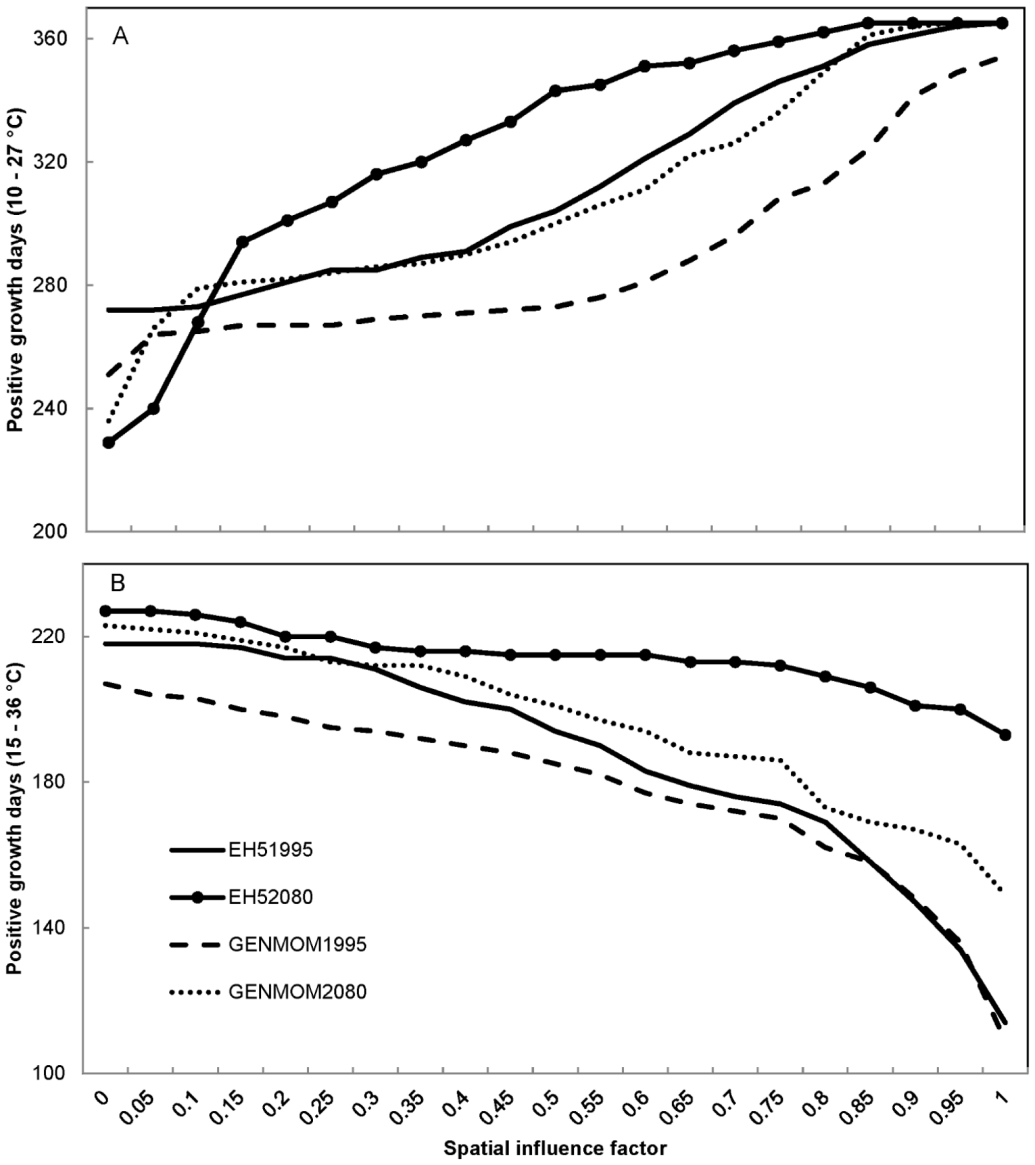
Predicted number of positive growth days for smallmouth bass *Micropterus dolomieu* (Panel A) and largemouth bass *Micropterus salmoides* (Panel B) in the Ozark National Scenic Riverways displayed by spatial influence factor values for two climate scenarios (EH5 and GENMOM) during 1995 and 2080.

**Figure 7:**
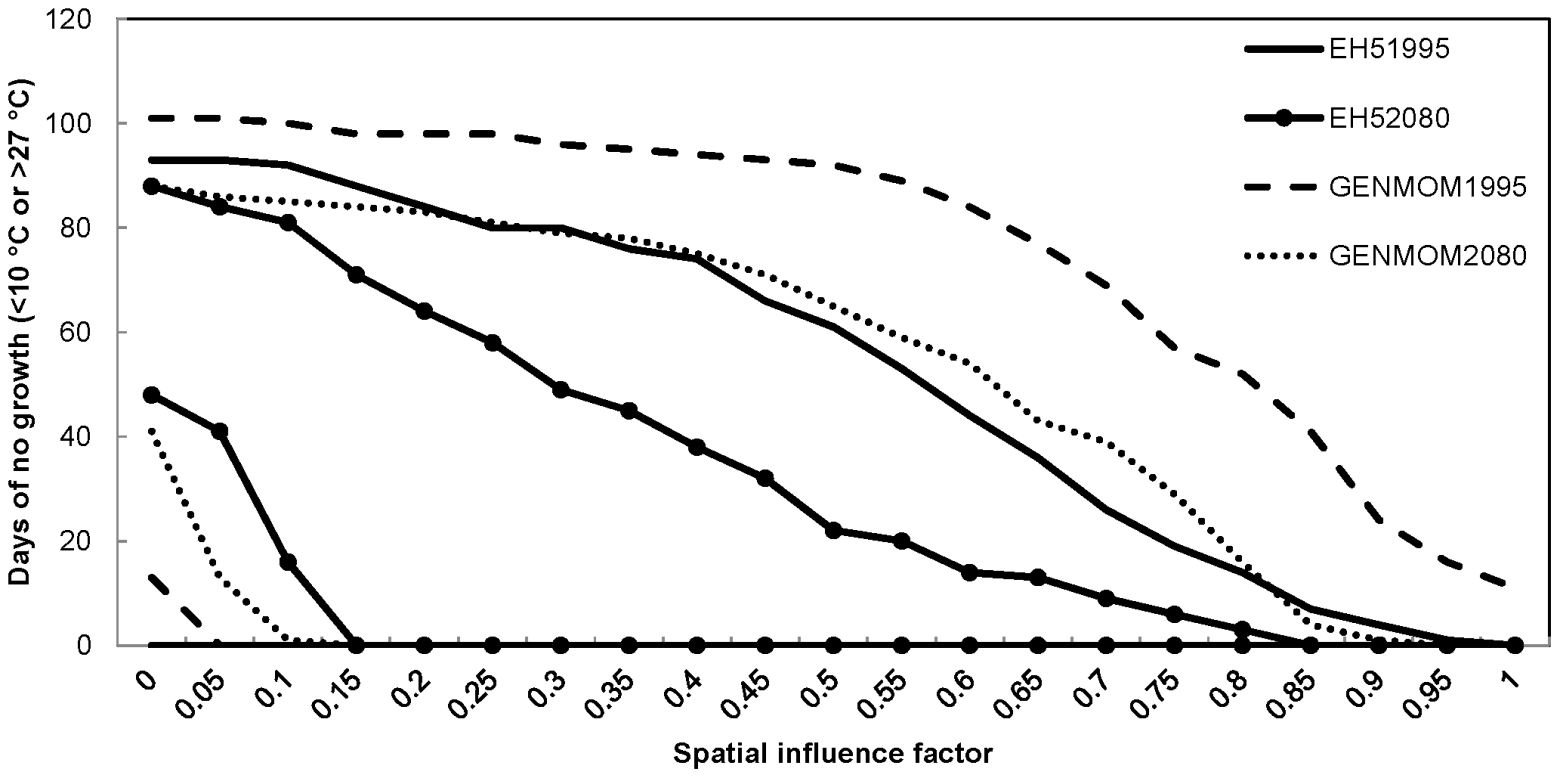
Predicted number of non-growing days for smallmouth bass *Micropterus dolomieu* in the Ozark National Scenic Riverways displayed by spatial influence factor values for two climate scenarios (EH5 and GENMOM) during 1995 and 2080. Days <10°C (too cold for growth) are displayed in the lower left corner and days >27°C (too warm for growth) are displayed as the top set of lines.

**Figure 8:**
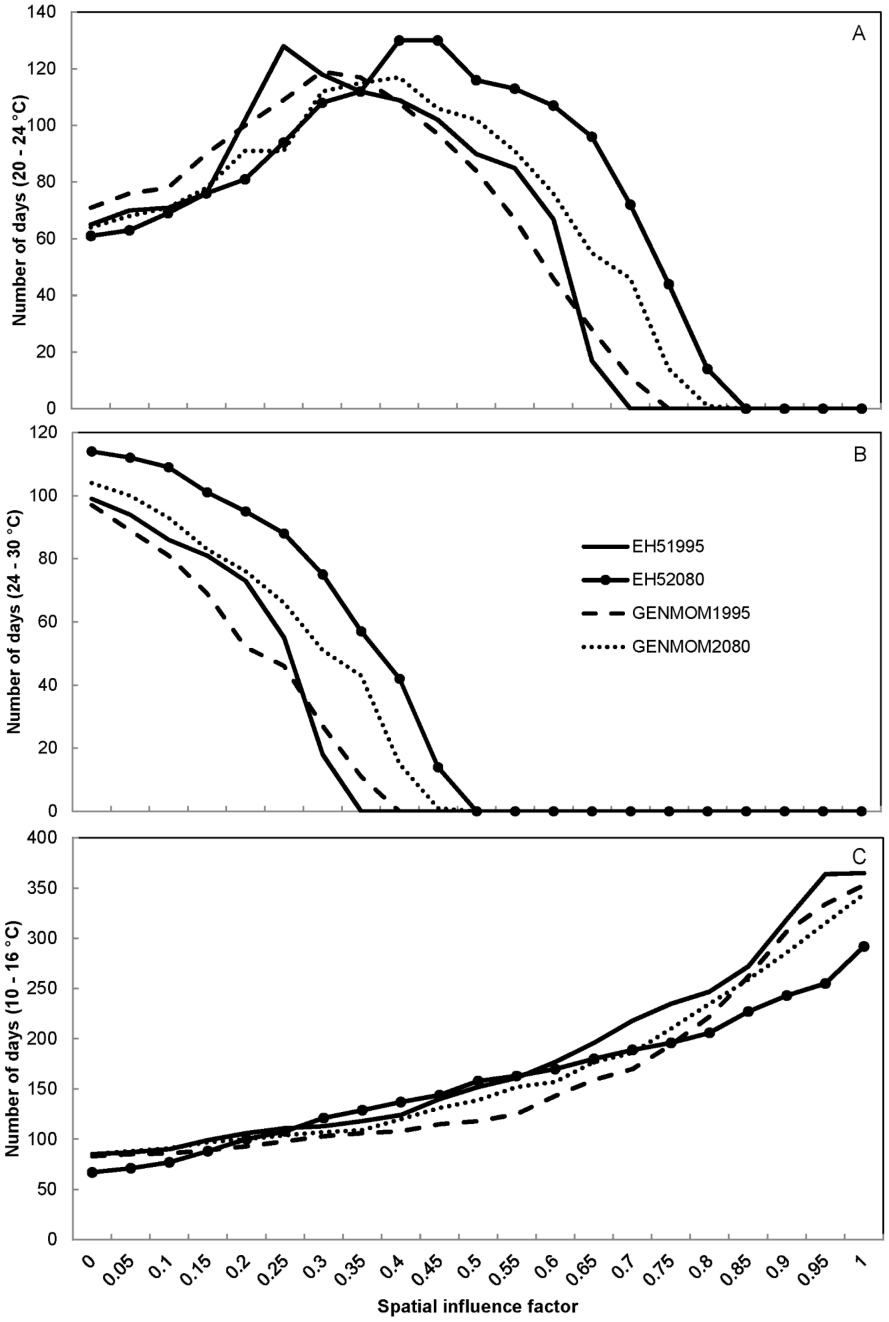
Predicted number of optimal growth days for smallmouth bass *Micropterus dolomieu* (Panel A), largemouth bass *Micropterus salmoides* (Panel B), and Ozark hellbenders *Cryptobranchus alleganiensis bishopi* (Panel C) in the Ozark National Scenic Riverways displayed by spatial influence factor values for two climate scenarios (EH5 and GENMOM) during 1995 and 2080.

We observed similar trends among our simulated water temperature results regardless of climate scenario. The number of positive growth days for smallmouth bass increased with increasing SIF values (more groundwater) and plateaued near SIF values of 0.85 for most simulations (Figure 6A). Both climate models predicted an increase in the number of positive growth days from 1995 to 2080 for smallmouth bass at locations with SIF values greater than 0.15, with as many as 39 more days of growth per year (13% increase) at an SIF value of 0.50 in the EH5 simulation. These patterns were explained by the reduced number of days too cold for smallmouth bass growth at moderate to high (>0.15) SIF values and the increased number of days too warm for growth at low (<0.15) SIF values (Figure 7). The GENMOM simulations indicated a similar trend to the EH5 simulations, but there were on average 8% fewer positive growth days across SIF values above 0.15 predicted for the year 2080 than predicted by the EH5 simulations. Based on the 1995 simulations, smallmouth bass were expected to experience the maximum number of optimal growth days in areas of river with either a 0.25 (EH5) or 0.30 (GENMOM) SIF value (Figure 8A). However, by 2080, both climate models predicted that those maxima will shift to areas of the river with greater groundwater influence (0.40 SIF value).

The predicted largemouth bass response to climate change scenarios was less complex than that of smallmouth bass. The number of positive growth days for largemouth bass was predicted to increase from 1995 to 2080 due to fewer cold-limiting days, with the EH5 model again predicting the largest increase (Figure 6B). The GENMOM model predicted that the number of positive growth days for largemouth bass will increase by 2080 from 7 to 37% (11 to 40 days per year), depending on SIF value. The EH5 model predicted large gains (up to 70% more days) in the number of positive growth days for high SIF value areas and smaller gains (as low as 3%) for low SIF value areas (Figure 6B). The number of optimal growth days for largemouth bass may increase by 20 days (21%) in areas with SIF values of zero to as much as 57 days a year (317%) in areas with SIF values near 0.3, based on the EH5 simulations for 1995 to 2080 (Figure 8B). The GENMOM simulations predicted a similar trend, but of lesser magnitude (5 to 33 days of additional optimal growth per year). The EH5 1995 model predictions indicate that in areas where SIF values are 0.15 or less, largemouth bass experience more days of optimal growth than do smallmouth bass. However, largemouth bass currently have three more days per year of optimal growth in areas where SIF = 0.15, and by 2080 that difference will increase to 23 days per year, which may have implications for interspecific competition (Figures 8A and B). All simulations indicated that areas with SIF values above 0.5 would not support any optimal growth days for largemouth bass due to water temperatures below 24°C.

The large range of temperatures at which hellbenders may experience positive growth resulted in negative growth days (< 3 or > 27°C) only in low spring influence area (SIF values < 0.15), and never for more than 48 days (13%) of a given year. The EH5 and GENMOM simulated trends were similar to each other for forecasted hellbender optimal growth days where both models predicted increasing optimal growth days with increasing SIF values (Figure 8C). Both models predicted fewer optimal growth days in 2080 than in 1995 for locations with either low (<0.25) or high (>0.85) SIF values, with up to a 20% reduction in optimal growth days in areas with SIF values of 1 (Figure 8C). The GENMOM and EH5 models predicted more optimal growth days in the future for mid-range SIF values (0.25–0.55 and 0.35 – 0.85; respectively) due to warmer winter temperatures; however, neither model predicted an increase of more than 27 days.

## Discussion

Our study provides a framework to document and predict fine-scale heterogeneous thermal conditions in lotic systems and complements other temperature modeling work typically conducted at larger spatial scales on surface-water systems. Our methods were based on easily obtainable data and accessible statistical techniques familiar to many biologists and managers. Characterization of thermal heterogeneity in the system allowed us to predict a probable shift in the spatial location of optimal growth temperatures available to two competing fish species under two climate change scenarios that otherwise might not have been identified by coarse-scale approaches.

### Temperature modeling approach

Scientists often consider multiple approaches to thermal modeling of stream networks and are faced with decisions regarding which predictor variables can and should be used [6]. These decisions sometimes result in the exclusion of groundwater streams from the dataset [7], post-hoc discussion of model limitations in relation to spatial variation caused by groundwater inputs [69], [71], or no mention of potential groundwater influence [2], [70]. We presented a novel approach to predict river temperatures using the spatial influence factor and readily available air temperature data to account for the combined effects of spatially important variables (e.g., groundwater input, stream size, amount of shading, tributary influence). Our modeling methods and the SIF could be easily adapted to use data from alternative methods for capturing broad-scale temperature variation data at fine resolutions such as distributed temperature sensing systems and thermal infrared imagery [72]–[76].

Our model indicated that groundwater had a significant effect on river water temperature and reduced water temperature variation; however, multiple linear regression models of water temperature in other streams not influenced by groundwater often perform well using only air temperature and flow variables [77]. In a study of Pennsylvania streams, groundwater controlled the stream-air temperature relationship and reduced the coefficient of variation in water temperature relative to a stream with less groundwater influence [78]. More complex responses of groundwater inputs to river water temperatures were observed during the summer in a California stream, where a 3.7 km long shallow spring branch resulted in delivery of water to the stream that was warmer than the receiving water at night and cooler during the day [12]. This phenomenon was explained by solar radiation warming water in the spring branch during the day that did not arrive to the river until night [12]. The shorter (≤ 1 km) and heavily shaded spring branches in the ONSR are likely affected less by solar radiation and are more consistent with groundwater temperature when they enter the stream.

We believe the SIF approach could be scaled-up and applied across any system given concurrent temperature data are available from multiple locations for its creation. We applied it on two rivers, but the concept of capturing spatial variation in a single metric using empirical data could apply to a system of any size. Our use of the spatial correction factor to interpolate SIF values between permanent temperature logger locations was useful for our objectives, but the SIF could be based solely on stationary temperature logger data if fine-scale resolution is not required. The SIF was based only on summer temperature maximums and although we had information on winter temperature extremes, we chose to use only the summer values because they showed more spatial variation. Using a separate SIF based on data from each season may better account for temporal relations of spatially important factors. This is especially important if seasonal relations between spatial factors and water temperature depart significantly from linearity.

Our temperature modeling approach had several assumptions and limitations. First, we chose to use a static value for groundwater temperature of 14°C. The influence of groundwater inputs may vary regionally based on groundwater temperatures and may change with future climatic conditions [79]. We observed a groundwater temperature at Alley Spring of approximately 14.2°C, which is 0.8°C higher than mean annual air temperature [59]. This was consistent with Mohseni & Stefan [37] who found that groundwater temperature is 1 – 2°C higher than mean annual air temperature in a given region. Temporal changes in groundwater temperature could be incorporated in the model to improve accuracy. Second, our model did not account for changes in river discharge and excluded temperature data from high water events (>75^th^ quartile discharge). Discharge and water temperature are closely linked and are often both used in predictive temperature models [80], but without data on runoff water temperature it would be difficult to reliably predict the effect of high water events on river temperature. Further, we assumed that high water events would likely result in acute responses to thermal conditions by aquatic organisms in the system and would therefore be less informative related to growth predictions than the static conditions we modeled. Finally, including multiple years of data to train and validate the model would incorporate greater seasonal and yearly variation. All of the data used in this study came from the calendar year 2012, which was one of the warmest and driest on record [59]. However, the warmest 5-day average period and coldest 5-day average period were within the range observed during the previous 30 years.

Our approach to predicting the effects of future climate on river temperature and the effects of those temperatures on the biota also have limitations. We chose to use two downscaled climate models that represent extremes within the A2 emissions scenario [60]. This approach was intended to display a range in possible response, but neither model may provide the best approximation of future air temperatures. Multi-model ensemble approaches have become common and may provide future climate data that are more robust to individual model assumptions and more accurately reflect future conditions [81]. Further, we only used models from the A2 emissions scenario which predicts a medium-high level of carbon dioxide emissions relative to other scenarios [82]. Thus, our simulations are likely intermediate to what might be expected based on scenarios that differ in global population projections, carbon emissions, and other factors. We also did not include predicted changes in groundwater temperature, spring or river discharge, or precipitation based on climate change scenarios. Changes in precipitation are predicted and may result in altered flow regimes and groundwater discharges [83]–[85]. Reduced groundwater discharge would be expected to result in warmer water temperatures in the summer and colder water temperatures in winter due to less advective heat flux between groundwater and surface water. Thus, the importance of the SIF variable would be reduced and water temperatures would likely be driven more by air temperature. Streams with less groundwater inflow than our system may experience increased thermal stress from the synchrony of low flow conditions and high temperatures because they lack the more static discharge provided by springs [80]. Finally, the air temperature values used in our climate simulations were based on one, 15-km grid location from within the study area. Topographic variation and other factors may result in air temperatures that differ from those we used in both the temperature model and climate simulations which may affect model accuracy.

### Ecological Relevance of Thermal Heterogeneity

Groundwater inflows, such as those we documented on the ONSR, exemplify the patchy nature of environmental conditions in lotic systems [86], [87]. Large magnitude springs in the ONSR altered water temperatures in the stream by up to 10°C during the warmest part of the year, effectively creating thermal patches which may affect the distribution of aquatic biota. For example in Tennessee, the Barrens topminnow *Fundulus julisia* is restricted to patchy environments created by groundwater springs [88]. Further, the physical processes that create patchy environments are linked to patterns in biocomplexity and may influence assemblage patterns of aquatic organisms, meta-population dynamics, and biogeochemical processes [89]–[91]. Unlike patchy environments created by hydrodynamic processes, thermal patches are dynamic in a seasonally predictable way. Thus, the patches may disappear during certain times of the year when water temperature is near groundwater temperature. We observed a lower magnitude influence of groundwater on river temperatures in the winter as compared to the summer. This is important for modeling efforts as we suspect springs that contribute 3% (summer) or 5% (winter) of total discharge will have minimal effects on overall river temperatures beyond a few hundred meters.

Our results show that spring magnitude is positively related to spring influence on river temperature and is the primary spatial driver of water temperature. Whitledge et al. [52] examined groundwater influence on river temperature during the summer and noted a positive relationship between increasing spring discharge and the distance needed for water temperatures to warm to mean daily temperatures. They also concluded that riparian shading was important in Ozark Streams, but that shading alone did not affect water temperature more than 2°C and was less important than groundwater influences.

Stream water temperature can be modeled at spatial scales ranging from 1–10 m patches [19], [92] to regional efforts covering thousands of km [9]. Our modeling efforts focused on a relatively fine-scale (10 s m grain size – 10 s km extent), which can provide area managers with detailed information on an entire system of interest and help identify the extent of potential thermal refugia for aquatic organisms. Our study did not identify thermal heterogeneity at very fine spatial scales (1 −10 m^2^ patches), but upwelling groundwater at that scale was used as thermal refuge by rainbow trout *Oncorhynchus mykiss* in Northwestern United States streams [19]. Despite the scale of observations, thermal refuges can also attract individual fish from great distances demonstrating the influence of groundwater beyond localized populations and single spatial scales, as evidenced by movements of >40 km by smallmouth bass (J. Westhoff, unpublished data).

### Thermal Heterogeneity and Biotic Response to Climate Change

Studies on the effects of climate change on fishes and other aquatic organisms have often focused on species distributional shifts at coarse spatial scales [36], [93]–[95]. However, less is known about the potential fine-scale distributional responses by populations or individuals and it is expected that fish will experience population-level effects prior to coarse-scale range shifts [96], [97]. Our study demonstrated that as the climate warms, smallmouth bass in groundwater-influenced rivers may need to occupy areas with greater spring influence to experience the maximum number of positive or optimal growth days. Failure to shift to optimal thermal conditions may result in decreased growth, as suggested for Great Lakes fishes [98]. Stream reaches with little spring-influence (SIF < 0.4) will become less thermally hospitable to smallmouth bass, while areas with moderate to high spring-influence (SIF > 0.4) will become more thermally suitable if air temperatures increase. We predicted that largemouth bass (a competitor of smallmouth bass) will also experience more optimal growth days in the future in areas of the river that were previously better thermally suited for smallmouth bass. Thus, as temperature regimes shift spatially, an increased competitive advantage may occur for species like largemouth bass in areas of low groundwater influence where resident smallmouth bass may experience more thermal stress and poorer growing conditions. Evidence for this already exists as largemouth bass were more successful than smallmouth bass in relatively warmer Ozarks streams [99]. Zweifel et al. [66] also predicted a similar trend for these species, but added that consumption demands for prey will favor largemouth bass over smallmouth bass in warmer systems. Species that are normally dominant in an ideal thermal regime can experience reductions in growth when exposed to subdominant species in altered thermal regimes as observed in juvenile steelhead *Oncorhynchus mykiss* and Sacramento pikeminnow *Ptychocheilus grandis* [100]. Other organisms not buffered from climate change may be similarly tied to a combination of higher energetic costs from metabolic processes and altered interspecific community interactions [34]. Thus, aquatic organisms may need to adapt to changing water temperature or possibly face reduced fitness.

Climate change may have positive effects on certain species if water temperatures increase the number of days that occur within the optimal thermal range for a species [101], [102]. Pease & Paukert [33] predicted that smallmouth bass in the midwest US (including Missouri Ozarks streams) would grow about 6% for every 1°C increase in water temperature, but will need 27% more food to reach that level of growth. Ideal growth conditions are maximized for hellbenders in areas with decreased temperature variability provided by large groundwater inputs. With warmer temperatures, the number of days too cold for hellbenders at moderate SIF values will decrease at a rate faster than the increase in the number of days that are too warm. This may provide marginal growth benefits for hellbenders occupying those sections of stream. However, we did not predict hellbenders in the ONSR to experience major shifts in distribution nor have significantly fewer optimal growth days (except in highly groundwater influenced habitats). Moreover, warmer temperatures may reduce the production of chytrid fungus *Batrachochytrium dendrobatidis* zoospores, which are produced in the greatest quantity at cold temperatures [103]. However, the thermal ranges we used to model hellbender response to climate change were not based on well-established and scientifically tested relationships, but rather our interpretation of available information. Thus, further study to refine the thermal ecology of hellbenders may better inform future models of hellbender response to climate change. .

Our study has implications for how climate change is assessed for fishes using thermal guilds at coarse-spatial scales. Fish thermal guilds are often used as a baseline or response variable to predict fish distributions based on current and future climate conditions [104], [105]. However, stream systems with fine-scale heterogeneous thermal conditions may allow for species from multiple thermal guilds to occupy the same short (<10 km) reach of stream. For instance, following the methods of Wehrly et al. [104] to classify thermal conditions in streams, we noted three separate thermal categories (cold-, cool-, and warm-stable) present in the ONSR. Coarse-scale climate change predictions based on fewer temperature loggers would not likely detect this potential variation and could misrepresent the effects of climate change on fish communities.

### Conclusions

Understanding the spatial and temporal variation in water temperature in lotic systems provides an opportunity to better explore ecological phenomena and predict biotic response to change. We developed a methodology for assessing thermal heterogeneity and predicting water temperatures in the present and future with fine-scale spatial resolution. The SIF metric allowed us to predict daily average water temperature for over 200 km of stream heavily influenced by groundwater. The resulting model was then used to predict daily average water temperature based on future climate simulations. Those results demonstrated that smallmouth bass will likely need to shift their distribution closer to springs to experience maximum optimal growth conditions, largemouth bass will experience improved growing conditions in all sections of the stream, and hellbender salamanders will experience little change except near springs. Although we demonstrated potential uses of these data with three species, additional research avenues exist, including combining temperature predictions with habitat availability to determine suitability for various aquatic species and communities. We hope others will build on our results to better refine stream temperature models in groundwater systems.

## Supporting Information

Appendix S1 **Major springs located in the Ozark National Scenic Riverways, their discharges from Mugel** ***et al.,*** **(2009), and spatial locations.** UTM coordinates are in Zone 15 North, NAD 1983.
(DOCX)

Appendix S2 **Temperature logger locations and range of record for the Ozark National Scenic Riverways.** UTM coordinates are in Zone 15 North, NAD 1983.
(DOCX)

Appendix S3 **Summary data for temperature loggers in the Ozark National Scenic Riverways during the year 2012.** Bolded values should be interpreted with caution because they are based on less than the full year of data.
(DOCX)

## References

[1] Benyahya L, Caissie D, St Hilaire A, Ouarda TBMJ, Bobée B (2007) A Review of Statistical Water Temperature Models. Can Water Resour J 32: 179–192.

[2] Jones LA, Muhlfeld CC, Marshall LC, McGlynn BL, Kershner JL (2013) Estimating thermal regimes of bull trout and assessing the potential effects of climate warming on critical habitats. River Res and Appl 30: 204–216.

[3] Hague MJ, Patterson DA (2014) Evaluation of statistical river temperature forecast models for fisheries management. N Am J Fish Manage 34: 132–146.

[4] Isaak DJ, Hubert WA (2001) A hypothesis about factors that affect maximum summer stream temperatures across montane landscapes. J Am Water Resour As 37: 351–366.

[5] Poole GC, Berman CH (2001) An ecological perspective on in-stream temperature: natural heat dynamics and mechanisms of human-caused thermal degradation. Environ Manage 27: 787–802. 11393314 10.1007/s002670010188

[6] Cassie D (2006) The thermal regime of rivers: a review. Freshwater Bio 51: 1389–1406.

[7] Al-Chokhachy R, Alder J, Hostetler S, Gresswell R, Shepard B (2013) Thermal controls of Yellowstone cutthroat trout and invasive fishes under climate change. Glob Change Biol 19: 3069–3081. 10.1111/gcb.12262 23687062

[8] DeWeber JT, Wagner T (2014) A regional neural network ensemble for predicting daily river water temperature. J Hydrol 517: 187–200.

[9] Isaak DJ, Luce CH, Rieman BE, Nagel DE, Peterson EE, et al (2010) Effects of climate change and wildfire on stream temperatures and salmonid thermal habitat in a mountain river network. Ecol Appl 20: 1350–1371. 20666254 10.1890/09-0822.1

[10] Herb WR, Stefan HG (2011) Modified equilibrium temperature models for cold-water streams. Water Resour Res 47: W06519.

[11] Kanno Y, Vokoun JC, Letcher BH (2014) Paired stream-air temperature measurements revel fine-scale thermal heterogeneity within headwater brook trout stream networks. River Res and Appl 30: 745–755.

[12] Nichols AL, Willis AD, Jeffres CA, Deas ML (2014) Water temperature patterns below large groundwater springs: management implications for coho salmon in the Shasta River, California. River Res and Appl 30: 442–455.

[13] Meinzer OE (1927) Large springs of the United States. United States Geological Survey Water Supply Paper:557. Obtained online from: http://pubs.er.usgs.gov/publication/wsp557.

[14] Smith H, Wood PJ (2002) Flow permanence and macroinvertebrate community variability in limestone spring systems. Hydrobiologia 487: 45–58.

[15] Reynolds WW, Casterlin ME (1979) Behavioral thermoregulation and the “Final Preferendum” paradigm. Am Zool 19: 211–224.

[16] Peterson JT, Rabeni CF (1996) Natural thermal refugia for temperate warmwater stream fishes. N Am J Fish Manage 16: 738–746.

[17] Matthews KR, Berg NH (1997) Rainbow trout responses to water temperature and dissolved oxygen stress in two southern California stream pools. J Fish Biol 50: 50–67.

[18] Torgersen CE, Price DM, Li HW, McIntosh BA (1999) Multiscale thermal refugia and stream habitat associations of Chinook salmon in northeastern Oregon. Ecol Appl 9: 301–319.

[19] Ebersole JL, Liss WJ, Frissell CA (2001) Relationship between stream temperature, thermal refugia and rainbow trout *Oncorhyncnus mykiss* abundance in arid-land streams in the northwestern United States. Ecol Freshw Fish 10: 1–10.

[20] Howell PJ, Dunham JB, Sankovich PM (2010) Relationships between water temperatures and upstream migration, cold water refuge use, and spawning of adult bull trout from the Lostine River, Oregon, USA. Ecol Freshw Fish 19: 96–106.

[21] Sada DW, Fleishman E, Murphy DD (2005) Associations among spring-dependent aquatic assemblages and environmental and land use gradients in a Mojave Desert range. Divers Distrib 11: 91–99.

[22] Martin RW, JT Petty (2009) Local stream temperature and drainage network topology interact to influence the distribution of smallmouth bass and brook trout in a central Appalachian watershed. J Freshwater Ecol 24: 497–508.

[23] Labbe TR, Fausch KD (2000) Dynamics of intermittent stream habitat regulate persistence of a threatened fish at multiple scales. Ecol Appl 10: 1774–1791.

[24] Brewer SK (2013) Groundwater influences on the distribution and abundance of riverine smallmouth bass, Micropterus dolomieu, in pasture landscapes of the Midwestern USA. River Res Appl 29: 269–278.

[25] Shuter BJ, MacLean JA, Fry FEJ, Regier HA (1980) Stochastic simulation of temperature effects on first year survival of smallmouth bass. T Am Fish Soc 109: 1–34.

[26] Garrett JW, Bennett DH, Frost FO, Thurow RF (1998) Enhanced incubation success for Kokanee spawning in groundwater upwelling sites in a small Idaho stream. N Am J Fish Manage 18: 925–930.

[27] Baxter CV, Hauer FR (2000) Geomorphology, Hyporheic exchange, and selection of spawning habitat by bull trout (Salvelinus confluentus). Can J Fish Aquat Sci 57: 1470–1481.

[28] Power G, Brown RS, Imhof JG (1999) Groundwater and fish – insights from northern North America. Hydrol Processes 13: 401–422.

[29] Petty JT, Hansbarger JL, Hunstman BM, Mazik PM (2013) Brook trout movement in response to temperature, flow, and thermal refugia within a complex Appalachian riverscape. T Am Fish Soc 144: 1060–1073.

[30] Hu Q, Wilson GD, Chen X, Akyuz A (2005) Effects of climate and landcover change on stream discharge in the Ozark Highlands, USA. Environ Model Assess 10: 9–19.

[31] Palmer MA, Lettenmaier DP, Poff NL, Postel SL, Richter B, et al (2009) Climate change and river ecosystems: protection and adaptation options. Environ Manage 44: 1053–1068. 19597873 10.1007/s00267-009-9329-1

[32] Pörtner HO, Knust R (2007) Climate change affects marine fishes through the oxygen limitation of thermal tolerance. Science 315: 95–97. 17204649 10.1126/science.1135471

[33] Pease AA, Paukert CP (2014) Potential impacts of climate change on growth and prey consumption of stream-dwelling smallmouth bass in the central United States. Ecol Freshw Fish 23: 336–346.

[34] Poloczanska ES, Hawkins SJ, Southward AJ, Burrows MT (2008) Modeling the response of populations of competing species to climate change. Ecology 89: 3138–3149. 10.1890/07-1169.1 31766801

[35] Rahel FJ, Olden JD (2008) Assessing the effects of climate change on aquatic invasive species. Conserv Biol 22: 521–533. 18577081 10.1111/j.1523-1739.2008.00950.x

[36] Eaton JG, Scheller RM (1996) Effects of climate warming on fish thermal habitat in streams of the United States. Limnol Oceanogr 41: 1109–1115.

[37] Mohseni O, Stefan HG (1999) Stream temperature/air temperature relationship: a physical interpretation. J Hydrol 218: 128–141.

[38] Eby LA, Helmy O, Holsinger LM, Young MK (2014) Evidence of climate-induced range contractions in Bull Trout *Salvelinus confluentus* in a Rocky Mountain watershed, U.S.A.. PLOS ONE 9: e98812. 24897341 10.1371/journal.pone.0098812 PMC4045800

[39] Chu C, Jones NE, Mandrak NE, Piggott AR, Minns CK (2008) The influence of air temperature, groundwater discharge, and climate on the thermal diversity of stream fishes in southern Ontario watersheds. Can J Fish Aquat Sci 65: 297–308.

[40] Sinokrot BA, Stefan HG, McCormick JH, Eaton JG (1995) Modeling of climate change effects on stream temperatures and fish habitats below dams and near groundwater inputs. Climatic Change 30: 181–200.

[41] Kundzewicz ZW, Mata LJ, Arnell NW, Döll P, Jimenez B, et al (2008) The implications of projected climate change for freshwater resources and their management. Hydrolog Sci J 53: 3–10.

[42] Lucas LK, Gompert Z, Ott JR, Nice CC (2009) Geographic and genetic isolation in spring-associated *Eurycea* salamanders endemic to the Edwards Plateau region of Texas. Conserv Genet 10: 1309–1319.

[43] Carmona-Catot G, Magellan K, Garćia-Berthou E (2013) Temperature-specific competition between invasive mosquitofish and an endangered Cyprinodontid fish. PLOS ONE 8: e54734. 23382951 10.1371/journal.pone.0054734 PMC3555637

[44] Rieman BE, Isaak D, Adams S, Horan D, Nagel N, et al (2007) Anticipated climate warming effects on bull trout habitats and populations across the interior Columbia River basin. T Am Fish Soc 136: 1552–1565.

[45] Rahel FJ, Bierwagen B, Taniguchi Y (2008) Managing aquatic species of conservation concern in the face of climate change and invasive species. Conserv Biol 22: 551–561. 18577084 10.1111/j.1523-1739.2008.00953.x

[46] Ormerod SJ (2009) Climate change, river conservation and the adaptation challenge. Aquat Conserv 19: 609–613.

[47] Mugel DN, Richards JM, Schumacher JG (2009) Geohydrologic investigations and landscape characteristics of areas contributing water to springs, the Current River, and Jacks Fork, Ozark National Scenic Riverways, Missouri. U. S. Geological Survey Scientific Investigations Report 2009–5138: 80 p.

[48] Strahler AN (1957) Quantitative analysis of watershed geomorphology. EOS T Am Geophys Un 38: 913–920.

[49] Peterson JT, Rabeni CF (2001) Evaluating the physical characteristics of channel units in an Ozark stream. T Am Fish Soc 130: 898–910.

[50] Panfil MS, Jacobson RB (2001) Relations among geology, physiography, land use, and stream habitat conditions in the Buffalo and Current River systems, Missouri and Arkansas. USGS/BRD/BSR-2001-0005, 111 p.

[51] Lloyd CD (2010) Spatial data analysis: an introduction for GIS users. Oxford: Oxford University Press. 206 p.

[52] Whitledge GW, Rabeni CF, Annis G, Sowa SP (2006) Riparian shading and groundwater enhance growth potential for smallmouth bass in Ozark streams. Ecol Appl 16: 1461–1473. 16937811 10.1890/1051-0761(2006)016[1461:rsageg]2.0.co;2

[53] Jeppesen E, Iversen TM (1987) Two simple models for estimating daily mean water temperatures and diel variations in a Danish low gradient stream. Oikos 49: 149–155.

[54] Jourdonnais JH, Walsh RP, Prickett F, Goodman D (1992) Structure and calibration strategy for a water temperature model of the lower Madison River, Montana. Rivers 3: 153–169.

[55] Pilgrim JM, Fang X, Stefan HG (1998) Stream temperature correlations with air temperatures in Minnesota: implications for climate warming. J Am Water Resour As 34: 1109–1121.

[56] Bartholow JM (1989) Stream temperature investigations: field and analytic methods. Instream Flow Information Paper No. 13. U.S. Fish and Wildlife Service Biological Report 89(17). 139 p.

[57] Bogan T, Mohseni O, Stefan HG (2003) Stream temperature-equilibrium temperature relationship. Water Resour Res 39(9): 1245.

[58] Erickson TR, Stefan HG (2000) Linear air/water temperature correlations for streams during open water periods. J Hydrol Eng 5: 317–321.

[59] NOAA, National Climatic Data Center. http://www.ncdc.noaa.gov/ Accessed 6/4/2014.

[60] Hostetler SW, Alder JR, Allan AM (2011) Dynamically downscaled climate simulations over North America: methods, evaluation and supporting documentation for users. U. S. Geological Survey Open-File Report 2011–1238, 64 p.

[61] Winemiller KO, Flecker AS, Hoeinghaus DJ (2010) Patch dynamics and environmental heterogeneity in lotic ecosystems. J N Am Benthol Soc 29: 84–99.

[62] Schramm HL Jr, Armstrong ML, Fedler AJ, Funicelli NA, Green DM, et al (1991) Sociological, economic, and biological aspects of competitive fishing. Fisheries 16(3): 13–21.

[63] Whitledge GW, Hayward RS, Rabeni CF (2002) Effects of temperature on specific daily metabolic demand and growth scope of sub-adult and adult smallmouth bass. J Freshwater Ecol 17: 353–361.

[64] Mohler HS (1966) Comparative seasonal growth of the largemouth, spotted, and smallmouth bass. Master’s Thesis. University of Missouri, Columbia.

[65] Struber RJ, Gebhart G, Maughan OE (1982) Habitat suitability index models: largemouth bass. United States Department of the Interior, Fish and Wildlife Service Report. FWS/OBS-82/10.16. 32 p.

[66] Zweifel RD, Hayward RS, Rabeni CF (1999) Bioenergetics insight into black bass distribution shifts in Ozark Border Regions streams. N Am J Fish Manage 19: 192–197.

[67] Hutchinson VH, Hill LG (1976) Thermal selection in the hellbender, *Cryptobranchus alleganiensis*, and the mudpuppy, *Necturus maculosus* . Herpetologica 32: 327–331.

[68] Jeong DI, Daigle A, St-Hilaire A (2013) Development of a stochastic water temperature model and projection of future water temperature and extreme events in the Ouelle River basin in Québec, Canada. River Res Appl 29: 805–821.

[69] Ouellet V, Secretan Y, St-Hilaire A, Morin J (2014) Daily averaged 2D water temperature model for the St. Lawrence River. River Res Appl 30: 733–744.

[70] Ahmadi-Nedushan B, St.Hilaire A, Ouarda TBMJ, Bilodeau L, Robichaud E, et al (2007) Predicting river water temperatures using stochastic models: case study of the Moisie River (Québec, Canada). Hydrol Processes 21: 21–34.

[71] Imholt C, Soulsby C, Malcolm IA, Hrachowitz M, Gibbins CN, et al (2013) Influence of scale on thermal characteristics in a large montane river basin. River Res Appl 29: 403–419.

[72] Cherkauer KA, Burges SJ, Handcock RN, Kay JE, Kampf SK, et al (2005) Assessing satellite-based and aircraft-based thermal infrared remote sensing for monitoring Pacific Northwest river temperature. J Am Water Resour As 41: 1149–1159.

[73] Madej MA, Currens C, Ozaki V, Yee J, Anderson DG (2006) Assessing possible thermal rearing restrictions for juvenile coho salmon (*Oncorhynchus kisutch*) through thermal infrared imaging and in-stream monitoring, Redwood Creek, California. Can J Fish Aquat Sci 63: 1384–1396.

[74] Selker J, van de Giesen N, Westhoff M, Luxemburg W, Parlange M (2006) Fiber optics opens window on stream dynamics. Geophys Res Lett 33: L24401.

[75] Westhoff MC, Savenije HHG, Luxemburg WMJ, Stelling GS, van de Giesen NC, et al (2007) A distributed stream temperature model using high resolution temperature observations. Hydrol Earth Syst Sc 11: 1469–1480.

[76] Webb BW, Hannah DM, Moore RD, Brown LE, Nobilis F (2008) Recent advances in stream and river temperature research. Hydrol Processes 22: 902–918.

[77] Neumann DW, Rajagopalan B, Zagona EA (2003) Regression model for daily maximum stream temperature. J Environ Eng-ASCE. 129: 667–674.

[78] O’Driscoll MA, DeWalle DR (2006) Stream-air temperature relations to classify stream-groundwater interactions in a karst setting, central Pennsylvania, USA. J Hydro 329: 140–153.

[79] Taylor CA, Stefan HG (2009) Shallow groundwater temperature response to climate change and urbanization. J Hydro 375: 601–612.

[80] Arismendi I, Safeeq M, Johnson SL, Dunham JB, Haggerty R (2013) Increasing synchrony of high temperature and low flow in western North American streams: double trouble for coldwater biota? Hydrobiologia 712: 61–70.

[81] Tebaldi C, Knutti R (2007) The use of multi-model ensemble in probabilistic climate projections. Philos T Roy Soc A 365: 2053–2075. 10.1098/rsta.2007.2076 17569654

[82] Intergovernmental Panel on Climate Change (2000) Summary for policymakers: emissions scenarios. Special report of Working Group III on the Intergovernmental Panel on Climate Change. 27p.

[83] Eckhardt K, Ulbrich U (2003) Potential impacts of climate change on groundwater recharge and streamflow in a central European low mountain range. J Hydro 284: 244–252.

[84] Nohara D, Kitoh A, Hosaka M, Oki T (2006) Impact of climate change on river discharge projected by multimodel ensemble. J Hydrometeorol 7: 1076–1089.

[85] Jyrkama MI, Sykes JF (2007) The impact of climate change on spatially varying groundwater recharge in the grand river watershed (Ontario). J Hydro 338: 237–250.

[86] Townsend CR (1989) The patch dynamics concept of stream community ecology. J N Am Benthol Soc 8: 36–50.

[87] Thorp JH, Thoms MC, Delong MD (2006) The riverine ecosystem synthesis: biocomplexity in river networks across space and time. River Res Appl 22: 123–147.

[88] Williams JD, Etnier DA (1982) Description of a new species, *Fundulus julisia*, with a redescription of *Fundulus albolineatus* and a diagnosis of the subgenus Xenisma (Teleostei: Cyprinodontidae). Occas Pap Mus Nat Hist, University of Kansas 102: 1–20.

[89] Winemiller KO, Taylor DH (1987) Predatory behavior and competition among laboratory-housed largemouth and smallmouth bass. Am Midl Nat 117: 148–166.

[90] McClain ME, Boyer EW, Dent CL, Gergel SE, Grimm NB, et al (2003) Biogeochemical hot spots and hot moments at the interface of terrestrial and aquatic systems. Ecosystems 6: 301–312.

[91] Tonolla D, Wolter C, Ruhtz T, Tockner K (2012) Linking fish assemblages and spatiotemporal thermal heterogeneity in a river-floodplain landscape using high-resolution airborne thermal infrared remote sensing and in-situ measurements. Remote Sens Environ 125: 134–146.

[92] Schmidt C, Bayer-Raich M, Schirmer M (2006) Characterization of spatial heterogeneity of groundwater-stream water interactions using multiple depth streambed temperature measurements at the reach scale. Hydrol Earth Syst Sci 10: 849–859.

[93] Mohseni O, Stefan HG, Eaton JG (2003) Global warming and potential changes in fish habitat in U.S. streams. Climate Change 59: 389–409.

[94] Ficke AD, Myrick CA, Hansen LJ (2007) Potential impacts of global climate change on freshwater fisheries. Rev Fish Biol Fisher 17: 581–613.

[95] Comte L, Buisson LM, Daufresne M, Grenouillet G (2013) Climate-induced changes in the distribution of freshwater fish: observed and predicted trends. Freshwater Biol 58: 625–639.

[96] King JR, Shuter BJ, Zimmerman AP (1999) Empirical links between thermal habitat, fish, and climate change. T Am Fish Soc 128: 656–665.

[97] Ayllón D, Nicola GG, Elvira B, Parra I, Almodóvar A (2013) Thermal carrying capacity for a thermally-sensitive species at the warmest edge of its range. PLoS ONE 8: e81354. 24282584 10.1371/journal.pone.0081354 PMC3840006

[98] Hill DK, Magnuson JJ (1990) Potential effects of global climate warming on the growth and prey consumption of Great Lakes fishes. T Am Fish Soc 119: 265–275.

[99] Sowa SP, Rabeni CF (1995) Regional evaluation of the relation of habitat to distribution and abundance of smallmouth bass and largemouth bass in Missouri streams. T Am Fish Soc 124: 240–251.

[100] Reese C, Harvey BC (2002) Temperature-dependent interactions between juvenile steelhead and Sacramento pikeminnow in laboratory streams. T Am Fish Soc 131: 599–606.

[101] Lehtonen H (1996) Potential effects of global warming on northern European freshwater fish and fisheries. Fisheries Manag Ecol3: 59–71.

[102] Daufresne M, Lengfellner K, Sommer U (2009) Global warming benefits the small in aquatic ecosystems. P Nat Acad Sci USA 106: 12788–12793. 10.1073/pnas.0902080106 PMC2722360 19620720

[103] Woodhams DG, Alford RA, Briggs CJ, Johnson M, Rollins-Smith LA (2008) Life-history trade-offs influences disease in changing climates: strategies of an amphibian pathogen. Ecology 89: 1627–1639. 18589527 10.1890/06-1842.1

[104] Wehrly KE, Wiley MJ, Seelbach PW (2003) Classifying regional variation in thermal regime based on stream fish community patterns. T Am Fish Soc 132: 18–38.

[105] Busiion L, Thuiller W, Lek S, Lim P, Grenouillet G (2008) Climate change hastens the turnover of stream fish assemblages. Glob Change Biol 12: 2232–2248.

