# Supplementary material for: Climate Change Simulations Predict Altered Biotic Response in a Thermally Heterogeneous Stream System
Source: PLoS One. 2014 Oct 30;9(10):e111438. doi: 10.1371/journal.pone.0111438 (PMC4214750; doi:10.1371/journal.pone.0111438)
Supplement: Appendix S1 — Major springs located in the Ozark National Scenic Riverways, their discharges from Mugel et al., (2009), and spatial locations. UTM coordinates are in Zone 15 North, NAD 1983. (DOCX) [file pone.0111438.s001.docx]

**Appendix S1**

| Spring | River | Discharge (m^3^/sec) | Spring discharge as % river discharge | River Meter | UTM Easting | UTM Northing |
| --- | --- | --- | --- | --- | --- | --- |
| Montauk* | Current | 1.38 | 99 | -5,200 | 616563 | 4146678 |
| Welch | Current | 2.83 | 57 | 19,959 | 626184 | 4139523 |
| Cave | Current | 0.47 | 9 | 32,845 | 633211 | 4136236 |
| Pulltite | Current | 2.49 | 32 | 41,070 | 634193 | 4132787 |
| Round | Current | 0.45 | 5 | 55,102 | 641462 | 4127168 |
| Cove | Current | 0.22 | 1 | 92,843 | 660050 | 4116908 |
| Blue | Current | 2.63 | 15 | 97,493 | 662932 | 4114906 |
| Gravel | Current | 0.37 | 2 | 118,023 | 672178 | 4110618 |
| Mill Creek | Current | 0.10 | 1 | 130,256 | 672671 | 4100675 |
| Bass Rock | Current | 0.97 | 5 | 135,975 | 676315 | 4097622 |
| Big | Current | 9.71 | 32 | 145,911 | 679065 | 4090963 |
| Blue | Jacks Fork | 0.30 | 27 | 4,254 | 621059 | 4101845 |
| Alley | Jacks Fork | 2.75 | 71 | 40,776 | 638906 | 4112708 |

Appendix S1. Major springs located along the Current and Jacks Fork Rivers in (or within close proximity of) the Ozark National Scenic Riverways, their discharges from Mugel *et al.,* (2009), and spatial locations. * indicates Montauk Spring was not included in the spring magnitude calculations. UTM coordinates are in Zone 15 North, NAD 1983.
