# Supplementary material for: Climate Change Simulations Predict Altered Biotic Response in a Thermally Heterogeneous Stream System
Source: PLoS One. 2014 Oct 30;9(10):e111438. doi: 10.1371/journal.pone.0111438 (PMC4214750; doi:10.1371/journal.pone.0111438)
Supplement: Appendix S2 — Temperature logger locations and range of record for the Ozark National Scenic Riverways. UTM coordinates are in Zone 15 North, NAD 1983. (DOCX) [file pone.0111438.s002.docx]

**Appendix S2**

| River | River Meter | Location | UTM Easting | UTM Northing | Start Date | Stop Date |
| --- | --- | --- | --- | --- | --- | --- |
| Current | 1 | Tan Vat | 618337.2 | 4145653.3 | 10/12/2011 | 3/14/2013 |
| Current | 12,203 | Cedar Grove | 623066.4 | 4142601.6 | 8/22/2011 | 3/14/2013 |
| Current | 20,966 | Welch | 626277.2 | 4138599.9 | 10/12/2011 | 3/28/2013 |
| Current | 24,519 | Akers | 627978.4 | 4137397.9 | 8/22/2011 | 3/14/2013 |
| Current | 39,517 | Pulltite Upstream | 635057.7 | 4132983.7 | 8/22/2011 | 3/14/2013 |
| Current | 42,648 | Pulltite Downstream | 634505.3 | 4131516.7 | 10/7/2011 | 3/14/2013 |
| Current | 54,195 | Round Spring | 640969.7 | 4127888.6 | 8/22/2011 | 3/14/2013 |
| Current | 75,183 | Jerktail | 649990.6 | 4121512.8 | 8/22/2011 | 5/29/2013 |
| Current | 84,568 | Two Rivers | 653300.6 | 4117510.2 | 10/13/2011 | 2/23/2013 |
| Current | 95,432 | Powder Mill | 661936.9 | 4116534.3 | 8/22/2011 | 5/29/2013 |
| Current | 98,439 | Blue Spring Downstream | 662516.5 | 4114080.5 | 10/6/2011 | 4/8/2013 |
| Current | 109,746 | Logyard | 665956.7 | 4109469.0 | 8/22/2011 | 4/8/2013 |
| Current | 117,837 | Paint Rock | 672036.8 | 4110762.3 | 8/22/2011 | 5/29/2013 |
| Current | 130,663 | Raft Yard | 672981.2 | 4100423.4 | 9/9/2011 | 4/8/2013 |
| Current | 145,504 | Big Spring Upstream | 678921.0 | 4091345.9 | 9/9/2011 | 5/29/2013 |
| Current | 146,599 | Big Spring Downstream | 679690.2 | 4090681.4 | 9/9/2011 | 5/29/2013 |
| Current | 159,915 | Cataract | 685964.8 | 4084624.8 | 9/8/2011 | 5/29/2013 |
| Current | 170,519 | Gooseneck | 683084.9 | 4076688.5 | 9/8/2011 | 9/28/2012 |
| Jacks Fork | 1 | Buck Hollow | 618646.4 | 4102067.4 | 8/28/2011 | 1/16/2013 |
| Jacks Fork | 15,182 | Rymers | 627929.7 | 4102681.4 | 8/22/2011 | 1/16/2013 |
| Jacks Fork | 30,127 | Bay Creek | 632838.9 | 4109273.4 | 8/22/2011 | 1/16/2013 |
| Jacks Fork | 39,686 | Alley Spring Upstream | 638059.9 | 4112529.1 | 8/22/2011 | 12/14/2012 |
| Jacks Fork | 40,776 | Alley Spring | 638883.1 | 4112713.1 | 2/24/2012 | 1/17/2013 |
| Jacks Fork | 41,899 | Alley Spring Downstream | 639488.1 | 4110129.4 | 9/30/2011 | 9/19/2012 |
| Jacks Fork | 42,530 | Keatons | 639855.5 | 4113911.1 | 1/19/2012 | 1/17/2013 |
| Jacks Fork | 58,441 | Shawnee Creek | 650746.5 | 4115481.6 | 8/22/2011 | 5/29/2013 |

Appendix S2. Temperature logger locations and range of record for the Ozark National Scenic Riverways. UTM coordinates are in Zone 15 North, NAD 1983.
