# Supplementary material for: Climate Change Simulations Predict Altered Biotic Response in a Thermally Heterogeneous Stream System
Source: PLoS One. 2014 Oct 30;9(10):e111438. doi: 10.1371/journal.pone.0111438 (PMC4214750; doi:10.1371/journal.pone.0111438)
Supplement: Appendix S3 — Summary data for temperature loggers in the Ozark National Scenic Riverways during the year 2012. Bolded values should be interpreted with caution because they are based on less than the full year of data. (DOCX) [file pone.0111438.s003.docx]

**Appendix S3**

| River | Location | Days of Record | Average Temperature (°C) | Standard Deviation | Maximum Temperature (°C) | Minimum Temperature (°C) |
| --- | --- | --- | --- | --- | --- | --- |
| Current | Tan Vat | 366 | 14.2 | 2.1 | 20.5 | 8.4 |
|  | Cedar Grove | 366 | 15.1 | 4.8 | 24.6 | 4 |
|  | Welch | 366 | 14.6 | 2.9 | 22.9 | 8.8 |
|  | Akers | **354** | **14.8** | **3.3** | 22.4 | 7.6 |
|  | Pulltite Upstream | 366 | 15.2 | 4.5 | 24.5 | 5.3 |
|  | Pulltite Downstream | 366 | 15 | 3.5 | 22 | 7.7 |
|  | Round Spring | 366 | 15.5 | 4.9 | 26.3 | 5.1 |
|  | Jerktail | 366 | 16.1 | 5.8 | 27.7 | 4.5 |
|  | Two Rivers | 366 | 16.4 | 6.1 | 28.3 | 4.4 |
|  | Powder Mill | 366 | 16.6 | 6.6 | 28.8 | 4.1 |
|  | Blue Spring Downstream | 366 | 16.3 | 5.9 | 27.3 | 4.8 |
|  | Logyard | 366 | 16.6 | 6.3 | 28.5 | 4.7 |
|  | Paint Rock | 366 | 16.9 | 6.6 | 30.5 | 3.7 |
|  | Raft Yard | 366 | 16.9 | 6.8 | 29.3 | 4.3 |
|  | Big Spring Upstream | 366 | 17.2 | 6.8 | 30 | 4.5 |
|  | Big Spring Downstream | 366 | 16.5 | 4.8 | 25.8 | 7.7 |
|  | Cataract | 366 | 16.7 | 5.3 | 26.8 | 7.2 |
|  | Gooseneck | **271** | **18.5** | **5.7** | 27.3 | 7.1 |
| Jacks Fork | Buck Hollow | 366 | 16.7 | 7.9 | 32 | 2.2 |
|  | Rymers | 366 | 17 | 7.6 | 31.5 | 2.6 |
|  | Bay Creek | 366 | 17 | 7.8 | 31.3 | 2.3 |
|  | Alley Spring Upstream | **349** | **17.6** | **7.2** | 30.8 | 3.4 |
|  | Alley Spring | **312** | **14.2** | **0.4** | 15.7 | 13.4 |
|  | Alley Spring Downstream | **260** | **15.6** | **3.7** | 21.9 | 7.1 |
|  | Keatons | **348** | **15.5** | **3.4** | 22.2 | 7.4 |
|  | Shawnee Creek | 366 | 16.2 | 5.7 | 27.5 | 4.9 |

Appendix S3. Summary data for temperature loggers in the Ozark National Scenic Riverways during the year 2012. Bolded values should be interpreted with caution because they are based on less than the full year of data.
